# Fluoxetine Regulates Ig Kappa Chain C Region Expression Levels in the Serum of Obsessive-Compulsive Disorder Patients: A proteomic Approach

**Published:** 2017

**Authors:** Mona Zamanian Azodi, Mostafa Rezaei Tavirani, Afsaneh Arefi Oskouie, Mostafa Hamdieh, Mohammad Kamran Derakhshan, Alireza Ahmadzadeh, Farid Zayeri, Naser Nejadi, Majid Rezaei Tavirani, Vahid Mansouri, Mohammad Rostami-Nejad, Reza Vafaee

**Affiliations:** a *Proteomics Research Center, Faculty of Paramedical Sciences, Shahid Beheshti University of Medical Sciences, Tehran, Iran. *; b *Psychosomatic Department, Taleghani Hospital, Faculty of Medicine, Shahid Beheshti University of Medical Sciences, Tehran, Iran. *; c *Faculty of Medicine, Iran University of Medical Sciences, Tehran, Iran. *; d *Research Institute for Gastroenterology and Liver Diseases, Gastroenterology and Liver Diseases Research Center, Shahid Beheshti University of Medical Sciences, Tehran, Iran. *; e *Physiotherapy Research Center, Shahid Beheshti University of Medical Sciences, Tehran, Iran .*

**Keywords:** Obsessive-Compulsive Disorder, Washing Subtype, Fluoxetine, Ig Kappa Chain C Region, Proteomics, Interactome Analysis

## Abstract

Obsessive-Compulsive Disorder (OCD) is one of the most common mental conditions. Proteome profiling may help identifying important proteins and finally shed lights to complexity of OCD underlying mechanisms. Here, by the application gel-based proteomic approach the proteome profile of patients with washing subtype of OCD before and after treatment with Fluoxetine (positive responders) are compared to healthy matched controls. However, only one of the differentially expressed proteins is examined and introduced in this paper. Proteomic analysis was done by the application of two-dimensional polyacrylamide gel electrophoresis (2-D PAGE), combined with (MALDI-TOF-TOF MS)-based. Furthermore, network analysis and biological annotation were handled by Cytoscape Plug-in and CluePedia. The proteome comparison between groups identified protein with the significant expression changes (p<0.05 and fold change ≥ 1.5). While the expression level of Ig Kappa Chain C Region is significantly decreased in OCD patients before any treatments, the trend is almost normalized after treatment with Fluoxetine in positive responders. In addition, interaction profile of IGKC shows that the interacting proteins may be affected as the expression pattern of IGKC changes in OCD patients. In conclusion, IGKC may be introduced as potential biomarker in our study; yet, investigation in bigger sample size and application of validation methods is a requirement.

## Introduction

The lifetime prevalence of OCD in general populations is about 1-3% (1). This severe mental condition influences the quality of life by the presence of obsessive thoughts and compulsive behavior. In addition, OCD is with different subtypes and co-morbidities (2). The American Psychiatric Association via the Diagnostic and Statistical Manual of Mental Disorders-version 5 (DSM-5) proposed OCD as a distinctive category with its own associated disorders (3). Many different neurochemical, neuroanatomic, genetic, neuroimmunology, and animal studies indicated that complex etiopathology of OCD is related to the various genetic and environmental factors (4). These factors can influence the phenotype of OCD. Many candidate genes with different polymorphisms have been reported for OCD risk (5). However, none of the candidate genes reached significant associations in OCD genome-wide and (eQTLS) studies. The heterogeneity of OCD renders clinical investigations, molecular analysis, and treatment responses. One of the solutions for this problem is to examine its subtypes (6). In fact, specific genes may contribute to different types of OCD (1). One of the important common subtypes of OCD is washing type (7). In this subtype, the patients suffer from obsessive thoughts of contaminations that urge to compulsive behavior and ritual washing (1). Washing symptoms are reported to be related to bilateral ventromedial prefrontal regions and right caudate nucleus hyperactivity (8). Molecular evaluation of this subtype shows that there are some possible contributing genes including *ESR1, DLGAP1, HTR3A-E, and GRIN2B *that are purposed for contamination and washing subtype (9-12). As mentioned earlier, most of molecular evaluations of OCD are in the genetic and genomic fields (13). No knowledge of proteome expression changes of OCD is available. Proteomics can be helpful to better understand disorder molecular etiology by monitoring the modified protein expression (14). 

The molecular changes may lead to dysfunction of many biological processes that are important for mechanism of OCD (15). On the other hand, there are many treatment options available for OCD. The combination of medication and psychotherapy is the most common. The first-line medication choice for OCD is the serotonin reuptake inhibitors (SSRIs) (16). One of the mostly applied SSRIs is Fluoxetine that is applied in many mental conditions including depression, panic, and OCD (17). The proteomic examination of OCD patients treated with medication can also provide essential knowledge of drug effects on proteome profile. In this way, the possible changes of OCD related proteins in response to the treatment as well as drug mechanisms and molecular side effects may be better explained. One of the important sources of detecting protein signatures is the serum (18). There are about thousands of proteins in serum (19). Hence, here, the proteome profile of OCD patients before and after treatment with Fluoxetine is screened and one of the important proteins almost regulated in the presence of treatment is introduced. 

## Experimental

*Sample Collection*

*Human Subjects*

Our washing model cases (35 women patients) with moderate severity without any previous treatments were diagnosed according to DSM-V and enrolled in our study from Taleghani Hospital, Tehran, Iran. The 20 healthy woman cases (without any previous family history of OCD) and patient samples were demographically matched. The inclusion criteria for patients selection was: Women with drug-naïve washing subtype of OCD cases aged between 20-30 years old and without any other kinds of mental disorders as well as no specific medication consumption for any other kinds of diseases. The selected healthy controls were among women without any previous history of mental disorder in themselves and in their family as well as no drug consumption during the study. he OCD patients were without any comorbidites and were not previously treated with any therapies. The patients were given written informed consents prior to their sampling. Two expert physiatrists assessed the clinical symptoms before and after treatment with medication by the application of Yale–Brown Obsessive Compulsive Scale. The first sampling from OCD patients was prior to Fluoxetine treatment. After 17 weeks of following patients treated with Fluoxetine (40-80 mg) range, the treatment resistance cases were excluded and only 16 samples were remained for further investigation. The patients that showed >35% reduction in Y-BOCS scores after the treatment were categorized as positive responders and their blood samples were recollected. Finally, the proteome of the 16 positive responders patients before and after treatment were compared with 20 controls.

**Figure.1:**
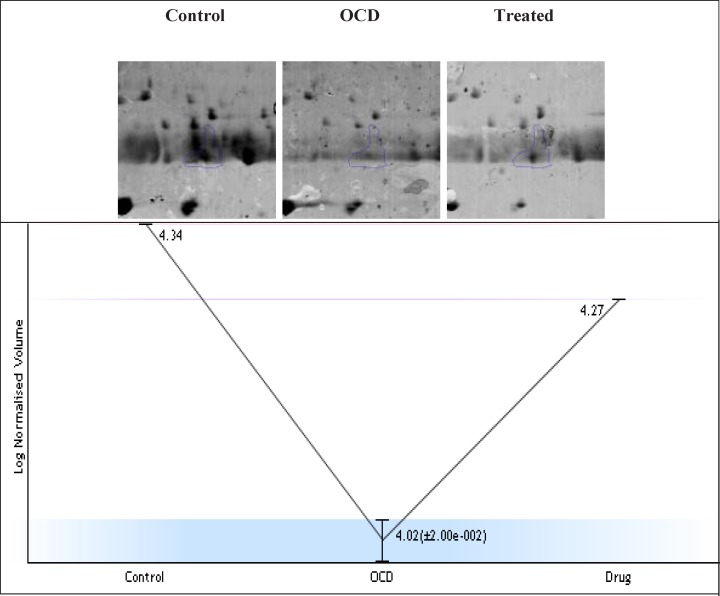
Expression changes of the selected spot in control, OCD and drug treated samples of 2DE Gel. The experiment was repeated three times. Fold change =2.1, p<0.05. The expression pattern of selected in the three gels with three technical repeats, the normalized volume changes from 4.34 to 4.27 in the samples. This pattern indicates that in OCD sample, the volume reduced to about 4.02 while it is almost went back to its normal value in treated sample by the value of 4.27. More details of alteration and compensation by the treatment are focused in the discussion part. The absolute amount of expression in normal condition is 21877. Protein expression in OCD condition is down regulated and reached to 10471. This change is equal to 2.1 fold change. In the presence of Fluoxetine, expression value is 18620. The approximate PI and MW of this spot are 5.58 and 11800 Da, respectively

**Figure.2:**
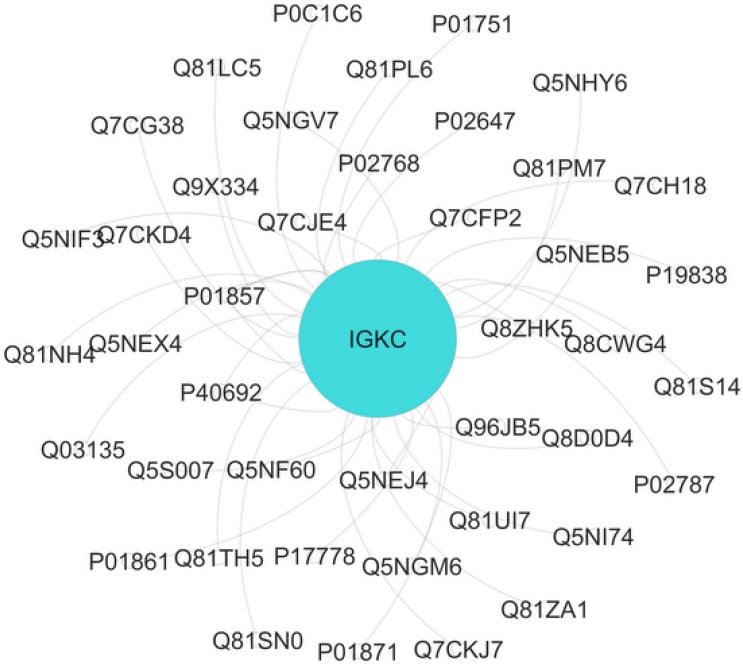
The interaction analysis of Ig kappa chain C region (IGKC) with neighbor proteins gray, obtained by Cytoscape. IGKC is identical with turquoise color. Nodes=43 Edges=45. Based on contribution at least two proteins in one molecular function (MF), four MF were obtained including sterol binding, phosphatidylcholine binding, immunoglobulin receptor binding, and copper ion binding for this interactome unit.

**Figure.3.**
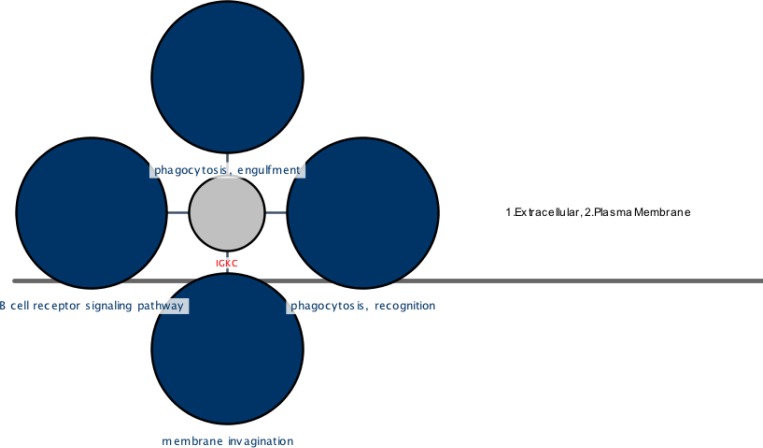
A cerebral view of the IGKC showing cellular locations and their related four top ranked biological processes (BP) colored in blue. These parts are coded as 1 (extracellular), 2 (plasma membrane) for IGKC. The phagocytosis, recognition, phagocytosis, engulfment, membrane invagination, and B cell receptor signaling pathway are the processes shown as dark blue circles and extracellular and plasma membrane are the locations of IGKC that are mentioned in the right position of the figure. The pvalue< 0.05

**Table.1:** The detailed information of IGKC spectra analysis by MASCOT Software. The protein shows significant identification score. The number of peptide sequences matched with IGKC in our protein query protein is 4

**Gene Name**	**Protein Name**	**Uniprot Code**	**Matching Score**	**Mass**	**Protein seq coverage**	**Peptide Matches**	**P- Value**
IGKC	Ig kappa chain C region	P01834	380	11773	48%	4	p≤ 0.05

**Table.2:** The detailed information correspond to biological process enrichments (gene ontology (GO)) of Figure 4. These terms are significantly associated with IGKC. GO terms and levels are provided Levels show the rank of terms in a biological process hierarchal term. The percentage of association genes are the percentage of IGKC contribution to that specific term. As it is clear, phagocytosis, recognition with 2.78 % relation, has the most significant contribution for our protein

**GO Term**	**GO Level**	**% Associated Genes**
phagocytosis,recognition	[4, 5, 6, 7]	2.78
phagocytosis,engulfment	[5, 6, 7]	1.67
membrane invagination	[4]	1.41
B cell receptor signaling pathway	[6, 8, 9, 10]	1.37

*Sample Preparation*

Blood collection was handled by venipuncture rout and using needle with gauge 2 °C. After clotting in the room temperature for 30 min, serum samples were completely separated by the centrifuging two times at 4 ºC with 2000 g and 10 min duration. Then the Eppendr of microtubes containing serum samples were kept at −80 °C until use. 

*Proteomic Analysis*

2D-electrophoresis materials were provided from GE HealthCare Life Sciences (http://www.gelifesciences.com) and SERVA Company (http://www.serva.de). Proteins from three individually pooled samples (healthy, drug-naive OCD patients, and positive responders to the treatment) were extracted using 2-DE Clean-Up Kit (GE Healthcare). For pooling procedure, 50 µL from each healthy patient and 62.5 µL for both untreated and treated OCD patients were pooled and the final volume reached 1cc for protein extraction for each groups separately. Protein concentrations were determined using 2-DE Quant Kit (GE Healthcare) and then 2DE procedure was performed with three-time replications for the samples. The first dimension, Isoelectric Focusing (IEF) separates proteins based on their pI. Prior to IEF, IPG strips were passively rehydrated in the presence of 1mg for 8 h. Then, IEF was carried out by the application of Bio-Rad PROTEAN IEF Cell, 11 cm nonlinear IPG with pH range 4-7 for 7.5 h. at 20 °C according to Bio-Rad Protocol. After that, IPG strips were then equilibrated for 30 min at room temperature in equilibration solution (Serva Kit). In the next step, separation was based on MW by the application of HPE FlatTop Tower (horizontal electrophoresis) using 2D HPE™ Double-Gel 12.5 % Kit (Serva Company) for about 3.5 h. After electrophoresis, the gels were stained by application of SERVA HPE^™^Coomassie^®^ Staining Kit according to the protocol and then scanned using a calibrated GS-800 densitometer  (Bio-Rad) scanner (20). Progenesis SameSpots Software as an image analyzer, detected protein expression changes by comparing normal, drug-naïve OCD patients, and OCD positive responders’ gels. In Samespots analysis, after automatically gel alignments and normalizations, the quantity amounts will assigned to each spots as logarithmic normalized volumes. A value of 1.5-fold increase or decrease was used as a cut-off. Statistically significant differences (*p*≤ 0.05) in spot intensities were identified using one-way ANOVA analysis. Finally, by the application of MALDI-TOF-TOF MS, the candidate spot was evaluated. In a way that, protein spots prior to treatment with trypsin, were de-stained and subjected to dithiotreitol (DTT) and iodoacetamide for reduction and alkylation, respectively. At the end, the extracted peptides were assessed by MS and the spectra were submitted to MASCOT (http://www.matrixscience.com) for protein identification. 

*Network Analysis*

Following to determine IGKC as a protein with alteration in its expression related to OCD and treatment, further investigation based on interaction analysis was carried out by Cytoscape *3.4.0-Milestone 2 *software (21). The application of PSICQUIC (Proteomics Standard Initiative Common QUery InterfaCe) and CluePedia analyzed IGKC interactome pattern and functional annotations, respectively. For functional enrichment analysis based on biological process the threshold selection was set to 4 terms and number and percent of genes were set to 1 and 1, respectively. IGKC and its annotation can be visualized as a pathway-like view with pre-defined cellular compartments. Cerebral view can provide defined parts including 1.Extracellular, 2.Plasma membrane, 3.Intra Cellular, 4.Nuclear Membrane, 5.Nucleus, 6.Transcription Factor Complex (22). 

## Results

Protein showed differences in accumulation levels in our study. The expression pattern and clustering of normal, drug-naïve OCD, and drug-treated samples are depicted in Figure 1. The mass spectrometry analysis showed that IGKC has associations with the serum proteome profile of OCD patients (see Table1 for more details)

The interaction and annotation analysis of IGKC is provided by the use of PSICQUIC (Proteomics Standard Initiative Common QUery InterfaCe) Cytoscape (Mentha Source) and CluePedia Plug-ins, respectively (see Figures 2, 3 and Table 2)

## Discussion

The complexity of obsessive-compulsive disorder made it as a challenging neuropsychiatric disorder with the respect to treatment selections (1). In this regard, molecular investigation can be helpful to detect certain biomarkers and to better understand of OCD underlying mechanisms and molecular responses in the presences of specific treatments (23). Moreover, studying protein expression changes as fundamental functional elements of an organism, can present novel findings about OCD related mechanisms (24). Here, proteomic study of drug-naïve OCD and positive responders to Fluoxetine samples is conducted. However, only one of the significant differentially expressed proteins in these samples is introduced and discussed in this paper. The nominated protein is Ig kappa chain C region (IGKC) that showed significant changes in our OCD sample. IGKC is located on chr2 and encodes constant domain of kappa-type light chains for antibodies. Its dysregulation has been suggested for some diseases such as Alzheimer’s disease as well as prognosis features in some types of solid cancers, specifically, breast cancer (25-27). As indicated in Figures 1, protein expression pattern of IGKC shows some changes in OCD patients (medication-free) and positive responders to Fluoxetine. The comparison of patients with normal conditions shows that 52% of protein expression is suppressed whereas in treated patients, this decrement is equal to 15%. The calculated amount indicated that Fluoxetine compensates 71% of protein expression down-regulation. In other words, the expression of IGKC is reduced in OCD patients before any treatments and then increased by Fluoxetine prescription. Meaning, IGKC expression is considerably returned to its normal expression level after treatment with Fluoxetine in positive responders. Apparently, Fluoxetine not only has effect on the protein structures but also may have effect on proteins’ expression levels. This shows that medication may have some positive regulatory effects on IGKC levels in serum. Additionally, there are many evidences about Fluoxetine properties and its ability to induce structural changes in the proteins structures (17, 28). It seems that continuous treatment of patients with Fluoxetine may lead to increment of this compensated value. In table1, the relevant information based on MASCOT analysis is presented in details, in which express that IGKC is significantly the recognized protein in our samples. As the expression pattern of IGKC alters in OCD patients, other proteins in interaction may be influenced. In this regard, as it is clear from Figure 2, the dysruglution of IGKC may affect IGKC protein neighbors. One of the important binding proteins in IGKC network is albumin, which was previously identified as a central protein in OCD proteomic pilot study by this group (2). Another considerable interacting protein is APOA1 that was also an important key protein in the mentioned study. Moreover, enrichment analysis showed in Figure 3, indicates that the processes may alter by the dysregulation of the correlated protein; yet, additional examination is required. Therefore, one of the differences between normal and patients with OCD is the expression changes of IGKC protein in serum. The data in Table 2 shows the important relation of phagocytosis, recognition process and IGKC indicate that, this process may be abnormally influenced as our protein expression changes in OCD patients’ serum. To our knowledge, this is the first study that links Ig kappa chain C region to the OCD risk. There are two novel findings of this study. First, significant down-regulation of IGKC in OCD patients indicates that this protein may be purposed as a possible candidate biomarker for OCD; however, validation studies are the requirement. Since, some patients are drug-resistant, this molecular marker may be used as discrimination factor for the two treated groups (sensitive and resistance responders to the drug) in early stage of the treatment. Sec, application of Fluoxetine may possess positive regulation effect on IGKC expression level in serum. Hence, introduction of IGKC as an abnormally expressed protein provided further understanding of OCD molecular basis and responses to Fluoxetine treatments. It is suggested that humoral immune system may have a role in OCD development; however, further monitoring is necessary. 

## Conclusion

The expression changes of IGKC may serve as potential target for OCD pharmacotherapy evaluations. However, further investigation and validation approaches can provide better resolution of IGKC behavior in OCD profile. Additionally, examining the interaction pattern of IGKC with other altered proteins in OCD and OCD drug-treated patients may provide additional knowledge about underlying mechanisms and effective treatment managements. Since this marker may be as a sensitive marker for disease onset as well as treatment, it may be consider as diagnostic and patient follow up probe. 
